# Insights into the Conformational Variability and Regulation of Human Nek2 Kinase

**DOI:** 10.1016/j.jmb.2008.12.033

**Published:** 2009-02-20

**Authors:** Isaac Westwood, Donna-Marie Cheary, Joanne E. Baxter, Mark W. Richards, Rob L.M. van Montfort, Andrew M. Fry, Richard Bayliss

**Affiliations:** 1 Section of Structural Biology, The Institute of Cancer Research, Chester Beatty Laboratories, 237 Fulham Road, London SW3 6JB, UK; 2 Cancer Research UK Centre for Cancer Therapeutics, The Institute of Cancer Research, Haddow Laboratories, 15 Cotswold Road, Sutton, Surrey SM2 5NG, UK; 3 Department of Biochemistry, University of Leicester, Leicester LE1 9HN, UK

**Keywords:** Nek2 kinase, cancer, X-ray crystallography, structure-based drug design

## Abstract

The Nek family of serine/threonine kinases regulates centrosome and cilia function; in addition, several of its members are potential targets for drug discovery. Nek2 is dimeric, is cell cycle regulated and functions in the separation of centrosomes at G2/M. Here, we report the crystal structures of wild-type human Nek2 kinase domain bound to ADP at 1.55-Å resolution and T175A mutant in apo form as well as that bound to a non-hydrolyzable ATP analog. These show that regions of the Nek2 structure around the nucleotide-binding site can adopt several different but well-defined conformations. None of the conformations was the same as that observed for the previously reported inhibitor-bound structure, and the two nucleotides stabilized two conformations. The structures suggest mechanisms for the auto-inhibition of Nek2 that we have tested by mutagenesis. Comparison of the structures with Aurora-A and Cdk2 gives insight into the structural mechanism of Nek2 activation. The production of specific inhibitors that target individual kinases of the human genome is an urgent challenge in drug discovery, and Nek2 is especially promising as a cancer target. We not only identify potential challenges to the task of producing Nek2 inhibitors but also propose that the conformational variability provides an opportunity for the design of Nek2 selective inhibitors because one of the conformations may provide a unique target.

## Introduction

The Nek family of serine/threonine kinases functions in cell cycle regulation (reviewed by O'Regan *et al*.^1^). The first member of this family, NIMA (never in mitosis A) kinase, was identified in a genetic screen and is an essential gene for entry into mitosis in *Aspergillus nidulans*.^2^ There are 11 kinases related to NIMA in the human genome, including Nek2, Nek6, Nek7 and Nek9, which are mitotic regulators.^1^ Nek2 has the highest sequence homology to the fungal NIMA kinase, although RNA interference knockdown of Nek2 does not significantly affect mitotic entry.^3^ Instead, the role of Nek2 is to initiate the separation of centrosomes at G2/M by phosphorylation of two components of the intercentriolar linkage, rootletin and C-Nap1.^4–6^ Nek2 is overexpressed in several cancer cell lines and primary breast cancers and has been identified as a potential drug target in cholangiocarcinoma.^7–10^ Nek2 dimerizes through a C-terminal coiled-coil domain that is essential for efficient cellular activity^11^ and is activated by phosphorylation on its activation loop at T175, which is reversed by PP1-mediated dephosphorylation.^12–14^

Although the structures of surprisingly few kinases have been captured in both active and inactive conformations, comparisons of these structures have identified the motifs that change conformation upon activation.^15,16^ The DFG and HRD motifs are highly conserved and contain catalytically vital aspartic acid residues. The activation loop is immediately C-terminal to the DFG motif, contributes to substrate binding and helps organize other motifs. The C-helix contains a conserved glutamic acid that forms a salt bridge with a conserved lysine residue within the nucleotide-binding cleft. Formation of this salt bridge is crucial for efficient catalysis.^15^ Many serine/threonine protein kinases are positively regulated by phosphorylation on their activation loop, and this event can help order the surrounding motifs through electrostatic interactions with arginines and/or lysines, particularly the conserved arginine of the HRD motif, an arginine/lysine on the activation loop (e.g., PKA) or the C-helix (e.g., Aurora-A). Phosphorylation is not sufficient to order the activation loop in some kinases that also require the binding of additional proteins, such as cyclins in the case of CDKs and TPX2 in the case of Aurora-A.^17,18^ These additional proteins bridge between the C-helix and the activation loop and promote an ordered active conformation of both elements of the kinase structure. In the case of CDKs, the partner cyclin is required to move the C-helix into an active conformation, whereas in the case of Aurora-A, the C-helix is constitutively in the active conformation and the partner TPX2 instead locks the activation loop into the active conformation. Interactions at the C-helix can also be inhibitory, as is the case for the Src family of tyrosine kinases, where an N-terminal SH3 domain and linker form an auto-inhibitory interaction that locks the C-helix into an inactive conformation reminiscent of inactive CDKs.^19,20^

Protein kinases are a major target for drug discovery programs, and several kinase inhibitors are now in routine use in the clinic.^21^ Most of these bind the nucleotide-binding cleft of the kinase, competing with ATP. Because there are more than 500 human kinases, all of which are similar around the nucleotide-binding cleft, the development of inhibitors that are potent against only one or a few kinases is a considerable challenge.^22^ The enzymatic activity of kinases is highly regulated. Structurally, this arises from at least two conformations of the kinase, enzymatically active or enzymatically inactive.^23,24^ It is more challenging to develop specific inhibitors that target the active kinase conformation (type I inhibitors) because kinases exhibit very similar active conformations with chemically similar nucleotide-binding clefts, although there are notable successes, such as PKB inhibitors.^25^ By contrast, structures of inactive kinases adopt diverse conformations, and the design of inhibitors that target inactive kinase conformations (type II inhibitors) is now well established.^26^ One drawback of targeting the inactive conformation is that it may be more likely that resistant mutations that do not affect the activity of the kinase will emerge.

The structure of a T175A point mutant of the human Nek2 kinase domain bound to the tyrosine kinase inhibitor SU-11652 has been previously solved at 2.2-Å resolution.^12^ In this structure, Nek2 adopts an inactive conformation that resembles the inactive conformation of Cdk2 or EGFR. Here, we describe further structures of Nek2 that show how the conformation of the inactive kinase depends on the bound ligand and give insights into the regulation of activity.

## Results

### The activation loop of Nek2 adopts several well-defined conformations dependent on the ligand bound to the nucleotide-binding cleft

**Fig. 1:**
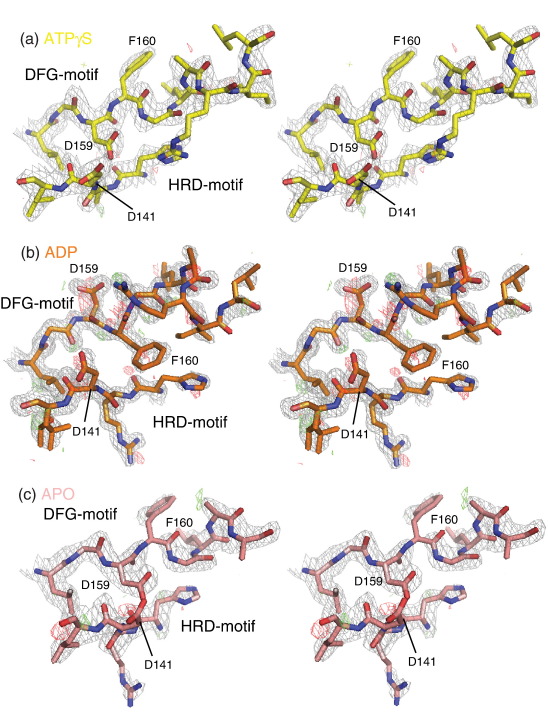
Electron density maps at the DFG and HRD motifs of Nek2. (a–c) 2*mF*_o_ − *DF*_c_ (gray) and *mF*_o_ − *DF*_c_ (green, red) SigmaA-weighted electron density maps contoured at 1.0σ, 2.5σ and − 2.5σ around (a) Nek2-T175A^ATPγS^, (b) Nek2^ADP^ and (c) Nek2-T175A^Apo^, respectively. Carbon atoms are shown in yellow, orange and pale pink in (a), (b) and (c), respectively. Oxygen atoms are shown in red, whereas nitrogen atoms are shown in blue. The same color scheme for stick representation is used in subsequent figures.

We have solved the crystal structures of apo-T175A Nek2 (Nek2-T175A^Apo^), ATPγS-bound T175A Nek2 (Nek2-T175A^ATPγS^) and ADP-bound wild-type Nek2 (Nek2^ADP^) kinase domains to 2.3, 2.4 and 1.55 Å, respectively. In this article, we refer to the previously solved structure of Nek2 bound to SU-116592 as Nek2-T175A^SU^. Overall, the electron density for all three new structures is excellent, and regions where the structures differed could be modeled with confidence (Fig. 1). Nek2-T175A^ATPγS^ is the most complete model, with only 8 of the 271 residues missing. The other two structures have 13 and 16 residues missing (Table 1).

**Fig. 2:**
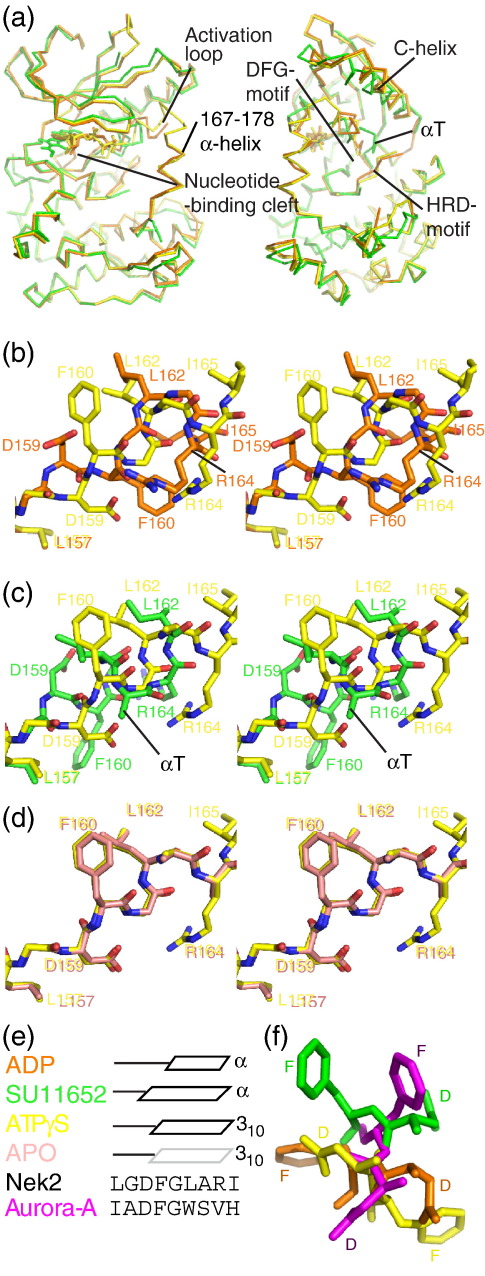
The DFG motif and activation loop adopt different conformations dependent on the bound ligand. (a) Superposition of Nek2-T175A^ATPγS^ (yellow), Nek2^ADP^ (orange) and Nek2-T175A^SU^ (green) protein structures shown as a ribbon in two orientations related by a 90° rotation about the *y*-axis. (b) Stereoview of Nek2-T175A^ATPγS^ (yellow carbon atoms) and Nek2^ADP^ (orange carbon atoms) superposition at the DFG motif. (c) Stereoview of Nek2-T175A^ATPγS^ (yellow carbon atoms) and Nek2-T175A^SU^ (green carbon atoms) superposition at the DFG motif. (d) Stereoview of Nek2-T175A^ATPγS^ (yellow carbon atoms) and Nek2-T175A^Apo^ (light pink carbon atoms) superposition at the DFG motif. (e) Schematic of the secondary structures adopted by the four Nek2 structures and the amino acid sequence surrounding the DFG motif in Nek2 and Aurora-A. (f) Superposition of three Nek2 conformations of the DFG motif together with the likely position adopted in the fully active conformation based on the Aurora-A/TPX2 structure (magenta). The orientation is that of panels (b) to (d) viewed from the bottom left to the top right.

The four structures exhibit overall very similar conformations (overall pairwise C^α^ RMSD = 0.20–0.95 Å; Fig. 2a). The most striking differences are located in the activation loop. Residues 167–178 form an α-helix in Nek2-T175A^ATPγS^, Nek2^ADP^ and Nek2-T175A^Apo^ but are disordered in Nek2-T175A^SU^. The mutation of T175 to alanine does not affect the structure of this helix; in addition, as this residue is not involved in any side-chain interactions in its unphosphorylated state, the mutation does not influence the structure. Strikingly, the region from residue 158 to residue 166, including the DFG motif (159–161) and part of the activation loop, adopts a completely different conformation in the Nek2-T175A^ATPγS^, Nek2^ADP^ and Nek2-T175A^SU^ structures (pairwise C^α^ RMSD = 2.8–4.5 Å; Fig. 2b–d). Nek2-T175A^Apo^ and Nek2-T175A^ATPγS^ are similar in this region, although the electron density for Nek2-T175A^Apo^ is weaker and the *B*-factors are higher, suggesting that this region is less well ordered in the apo kinase than in a ligand-bound kinase (Figs. 1c and 2d). In Nek2-T175A^SU^, this region forms an α-helix (αT) starting at D159, whereas in the other structures, the helical conformation starts at F160 or G161 and is a 3_10_-helix in Nek2-T175A^Apo^ and Nek2-T175A^ATPγS^ (summarized in Fig. 2e). All four structures superpose precisely at L157 and begin to diverge at G158. At D159, there is an approximately 180° difference in the orientation of the side chain between Nek2^ADP^ and Nek2-T175A^ATPγS^ (Fig. 2b and f). In Nek2-T175A^ATPγS^, the D159 side chain sits close to the HRD motif and forms hydrogen bonds with the main chains of D141 and G161 (Fig. 1a), whereas in Nek2^ADP^, the hydrogen bond is made with the side chain of K37. The side chain of F160 also shows a 180° flip between Nek2^ADP^ and Nek2-T175A^ATPγS^. In fact, F160 in Nek2^ADP^ has an orientation similar to D159 in Nek2-T175A^ATPγS^ and F160 in Nek2-T175A^ATPγS^ has an orientation similar to D159 in Nek2^ADP^ (Fig. 2f). F160 in Nek2^ADP^ sits close to the main chain of the HRD motif and interacts with the hydrophobic portion of the R164 side chain and the I165 side chain (Fig. 1b). F160 in Nek2-T175A^ATPγS^ packs against K37, L162, I84 and M86. It is remarkable that either of the D159 and F160 side chains can be accommodated in these two positions. In Nek2-T175A^SU^, these two side chains occupy yet another set of positions (Fig. 2f). The orientation of the D159 side chain in Nek2-T175A^SU^ is roughly halfway between that observed in the other two structures and forms a hydrogen bond with the L162 main chain. This hydrogen bond caps and presumably stabilizes the αT helix. In Nek2-T175A^SU^, the HRD motif is disordered and the side chain of F160 in Nek2-T175A^SU^ lies in the path followed by the main chain of the HRD motif in the other Nek2 structures and packs against L157, L124 and L142. None of the positions occupied by D159 and F160 in these three structures is what would be expected in an active conformation (Fig. 2f). In other words, Nek2 exhibits three examples of so-called DFG-out conformations. Indeed, the pocket that would be occupied by F160 in the active conformation is unoccupied in three of the structures and by an ethylene glycol molecule from the cryoprotectant in Nek2^ADP^. D159 is a minimum of 90° away from its position in an active conformation in all four structures. L162 from Nek2^ADP^ and Nek2-T175A^ATPγS^/Nek2-T175A^Apo^ fits into a pocket formed from L52, E55 and L59 of the C-helix and I83. In Nek2-T175A^SU^, the side chain of I165 fits into the equivalent pocket. The R164 side chain forms different contacts in all three structures and adopts very different positions. R164 interacts with H139 in Nek2-T175A^ATPγS^, with the β-phosphate of ADP in Nek2^ADP^ and with E55 in Nek2-T175A^SU^.

**Fig. 3:**
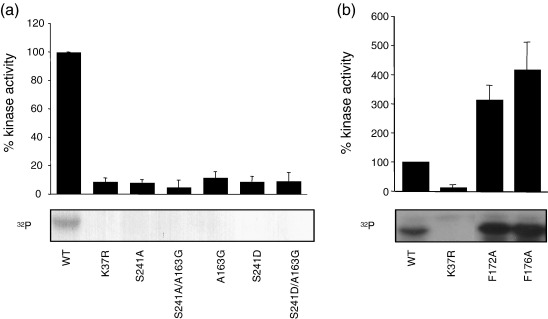
Mutations in the activation loop that increase or decrease Nek2 kinase activity. *In vitro* transcribed/translated wild-type and mutant Nek2 proteins were immunoprecipitated and assayed for kinase activity. (a) Mutation in the activation loop proximal to the DFG motif (A163G) or in the phospho-regulated residues (S241A, S241D) compared with wild type (WT) and kinase-dead mutant (K37R). (b) Mutations in the hydrophobic plug motif (F172A and F176A) compared with WT and K37R.

The region of the activation loop proximal to the DFG motif has been proposed to form an inhibitory helix (αT; Fig. 2a and c).^12^ If this were a feature of the inactive conformation that required unfolding for activity, then one would predict that disruption of the secondary structure would increase the activity of the kinase. We made an A163G mutation in the activation loop to disrupt the secondary structure and tested the activity of the mutant kinase (Fig. 3a). This mutation reduced the activity of the kinase to the same extent as a mutation in a catalytic residue (K37) or regulatory residue (S241).^12^ Thus, it is unlikely that conversion from the inactive to the active conformation relies purely on unfolding of this putative inhibitory helix.

### Coordination of nucleotide

**Fig. 4:**
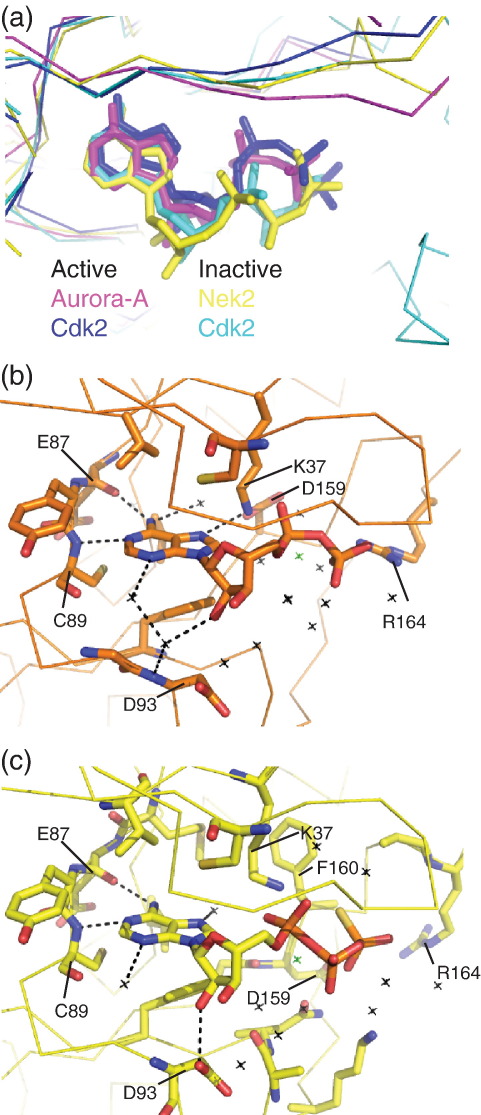
Network of interactions that loosely bind nucleotide to Nek2. (a) Superposition of nucleotide-binding clefts of two inactive kinase conformations [Nek2-T175A^ATPγS^ (yellow) and ATP-bound Cdk2 (cyan, Protein Data Bank code 1B39)] and two active kinase conformations [ADP-bound Aurora-A (magenta, Protein Data Bank code 1OL5) and ATP-bound Cdk2 (blue, Protein Data Bank code 1QMZ)]. (b) Structural representation of the interactions that bind ADP to inactive Nek2, including water molecules (black crosses) and a magnesium ion (green cross). (c) Structural representation of the interactions that bind ATPγS to inactive Nek2, including water molecules (black crosses) and a magnesium ion (green cross).

In common with other protein kinase structures, the nucleotide sits in the active-site cleft of Nek2 between the Gly-rich loop and β7/β8. The positioning of the nucleotide within the cleft resembles that found in other inactive kinase structures, in which the phosphates lie closer to the solvent-exposed side of the pocket than is observed in kinase active conformations. This is illustrated in Fig. 4a, where the nucleotides bound to active Aurora-A and Cdk2 are shown in magenta and blue, respectively, compared with inactive Cdk2 and Nek2, which are shown in cyan and yellow, respectively.

Both ADP and ATPγS are bound in the active-site pocket by a network of hydrogen bonds, electrostatic interactions and hydrophobic contacts (Fig. 4b and c). Most of these interactions are identical between the Nek2^ADP^ and Nek2-T175A^ATPγS^ structures. The hinge region of Nek2 forms two hydrogen bonds with the adenine base, the main-chain oxygen of Glu87 is an acceptor and the main-chain nitrogen of Cys89 is a donor. A network of water molecules links the adenine N^3^ and the ribose 2′ hydroxyl with the main-chain nitrogens of Gly92 and Asp93 and the Asp93 side chain. The Gly-rich loop (amino acids 15–20) contacts nucleotide phosphates directly through water molecules. A single magnesium ion bridges the α- and β-phosphates and bridges to the main chain of the DFG motif through water molecules. A clear difference between the Nek2^ADP^ and Nek2-T175A^ATPγS^ protein structures is the interactions formed by Arg164. In Nek2^ADP^, it forms a salt bridge with the β-phosphate of ADP, whereas in Nek2-T175A^ATPγS^, it interacts with Asp159 of the DFG motif (Figs. 2 and 4c).

### A crystal contact that resembles a hydrophobic plug forms an auto-inhibitory motif

**Fig. 5:**
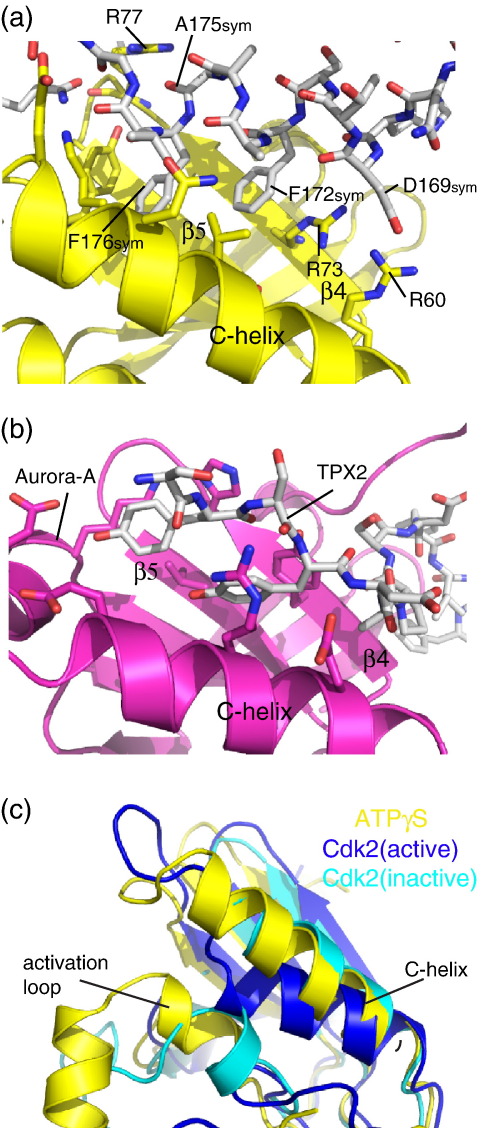
A crystal contact in Nek2 makes a hydrophobic plug–hydrophobic groove interaction. (a) Interactions at a crystal contact between two Nek2 molecules shown as yellow cartoon/stick for the hydrophobic groove-bearing symmetry-related partner and as sticks (gray carbon atoms) for the hydrophobic motif-bearing partner. (b) The hydrophobic groove–hydrophobic plug interaction between Aurora-A (magenta cartoon and carbon sticks) and TPX2 (white carbon sticks). (c) Superposition of Nek2-T175A^ATPγS^ (yellow), inactive Cdk2 (cyan) and cyclin-bound Cdk2 (blue).

In the three Nek2 structures described in this article, the activation loop forms a crystal contact with a hydrophobic groove between the C-helix and β4/β5 strands of an adjacent molecule (Fig. 5a). This contact bears a striking resemblance to the hydrophobic groove–hydrophobic plug interaction involved in AGC (protein kinases A, G and C) and Aurora kinase regulation (Fig. 5b, reviewed by Gold *et al*.^28^). The side chains of F172^sym^ and F176^sym^ pack into the hydrophobic groove. There are also interactions between D179^sym^ and R60/R79 and between the main chain of A176^sym^ and R77. There is no other crystal contact involving the C-helix. A key role for the hydrophobic plug is to increase the stability of the kinase, essentially by completing the hydrophobic core of the N-lobe (reviewed by Gold *et al*.^28^). Indeed, the crystal contact directs the ordering of the C-helix of Nek2^ADP^ and Nek2-T175A^ATPγS^ (all side chains ordered) compared with Nek2-T175A^SU^ (7 of 18 side chains disordered), which does not have the same crystal contact. The full-length Nek2 protein dimerizes through a C-terminal coiled-coil domain.^11^ To investigate whether this crystal contact might represent a physiological interaction in the Nek2 dimer, we made mutants in the full-length protein to disrupt the interface (F172A and F176A). The mutations resulted in a three- to fourfold increase in kinase activity, the effect opposite to that expected if this interaction activated the kinase. Moreover, because the crystal contact stabilizes an inactive conformation of the C-helix, this suggests that the crystal contact may mimic an auto-inhibitory interaction in the full-length protein.

## Discussion

Here, we describe three quite distinct structures of the kinase domain of the cell cycle-regulated human protein Nek2. When compared with the inhibitor-bound structure, T175A-Nek2^SU^, the activation loop exhibits a consistent difference in residues 167–178 influenced by crystal packing. The DFG motif and proximal region (residues 158–166) can adopt three conformations dependent on the ligand bound; in the absence of ligand, this region is less well ordered. This raises the question of how ligands might direct the different conformations of the DFG motif. One crucial difference between Nek2-T175A^SU^ and the other two ligand-bound structures is that SU-116592 buries a hydrophobic moiety deeper into the nucleotide-binding cleft. L162 forms a close van der Waals interaction with SU-116592, whereas in the other Nek2 structures, L162 is buried into a pocket in the underside of the C-helix. The SU-116592 ligand also prevents the interaction between D159 and K37 and does not present an alternative partner for K37. Instead, K37 forms a hydrogen bond with the side-chain hydroxyl of Y19, which adopts a rotamer that brings it under the Gly-rich loop. The main difference between the Nek2^ADP^ and Nek2-T175A^ATPγS^ protein–ligand contacts concerns R164, which interacts with the β-phosphate of ADP but interacts with the side chain of D159 in Nek2-T175A^ATPγS^. In Nek2^ADP^, D159 interacts with K37, and in both structures, K37 interacts with the α-phosphate of the nucleotide. The altered interactions of R164 and D159 (within and immediately C-terminal to the DFG motif) direct the altered conformation of the main chain.

In Nek2-T175A^SU^, the DFG motif and five following residues form an α-helix (αT), a feature that was proposed to be an inhibitory motif requiring unfolding in order to achieve the active conformation.^12^ The structures of Nek2 described in this article show that this region can adopt one of several conformations, and the precise conformation of the kinase is sensitive to the ligand occupying the nucleotide-binding cleft. Crucially, we found that in the Nek2-T175A^Apo^ structure, the activation loop appeared less ordered than in structures with ligand bound, and we have also observed that this region is disordered in apo-form crystals produced using ADP co-crystals soaked for 24 h into an ADP-free solution (data not shown). We conclude that much of the Nek2 structure forms a rigid framework that is characteristic of the inactive conformation, around which the variable regions organize. The variable regions include the DFG motif, activation loop and HRD motif. It appears that these regions become ordered by forming electrostatic interactions (e.g., E55, K37 and the ligand) and hydrophobic interactions with available partners (e.g., L52/E55/L59 pocket, HRD motif and hydrophobic surfaces of the ligand). That the rest of the structure is stable and that the activation loop can readily adopt one of several conformations call into question the assignment of this region as being auto-inhibitory. Moreover, mutations that disrupt the secondary structure in this region do not activate the kinase but reduce activity. Instead, it appears more likely that a structural element that is in a similar conformation in all four structures presents a barrier to a fully ordered structure and full activity. One candidate structural element is the C-helix, which is displaced from the position observed in active kinase conformations (Fig. 5c). Indeed, the C-helix adopts a position redolent of inactive Cdk2 and would similarly require a rotation of approximately 90° to move the catalytic glutamic acid (E55 in Nek2) into the active position (Fig. 5c). How could this structural transition occur? Although activation of the kinase requires phosphorylation on T175, the question remains as to whether this would be sufficient to stabilize the active conformation. There is no arginine or lysine residue in the C-helix in a suitable position to coordinate with phospho-T175 and direct its movement into an active position. Hence, movement of the helix may require a binding partner that bridges between the C-helix and the activation loop, as is the case for Cdk2/cyclin. In Nek2, this would involve the binding of a hydrophobic plug motif to the hydrophobic groove located at the C-helix. The hydrophobic motif at the crystal contact cannot fulfill this function and instead appears to form an auto-inhibitory interaction as demonstrated by the activating mutants F172A and F176A. An alternative hydrophobic motif partner may have to displace the F172/F176 motif, and this could result from a conformational change in the dimeric, full-length protein or binding of an additional protein factor. Further studies will be required to investigate the requirement of a hydrophobic motif–groove interaction for Nek2 regulation and the identity of the hydrophobic motif donor.

The fact that the DFG motif and activation loop can adopt several stable conformations is a possible hurdle to the rapid production of inhibitors that specifically target Nek2. For example, computational techniques, such as virtual screening, must be able to handle the conformational variability of the protein. Relatively small changes to hit compounds might induce stabilization of a different conformation, confusing structure–activity relationship analyses. On the other hand, the variability might be considered an opportunity as one of these conformations may provide the right template for a selective Nek2 inhibitor. Inhibitors that target the DFG-out conformation are perhaps the most promising in terms of selectivity.^24^ The conformation of Nek2^ADP^ most resembles the DFG-out conformation of tyrosine kinases recognized by selective inhibitors, such as imanitib, and there is a solvent-filled channel that connects the ADP-binding site to a hydrophobic pocket, occupied by an ethylene glycol molecule, that would be filled by the DFG motif phenylalanine in the active conformation. We predict that ligand–protein crystal structures will play a central role in a Nek2 drug discovery program. This endeavor will be greatly aided by our apo-form crystals that grow in suitable conditions for soaking experiments with a variety of ligands.

Another important consideration is whether it is possible to predict which other kinases can adopt multiple stable inactive DFG motif conformations. Two notable features of Nek2 may explain why the DFG motif can adopt very divergent conformations: First, it has a glycine N-terminal to the DFG motif, which is the point of divergence for the four Nek2 structures. Less than 10% of human kinases have a glycine in this position, giving rise to a low energetic barrier to transitions between different conformations of the DFG motif. This glycine residue has been proposed as an important determinant of flexibility by comparison of different tyrosine kinases, and the structures we present demonstrate how this glycine permits variability in different structures of the same kinase.^29^ Second, the inactive position of the C-helix leaves a large cavity for the activation loop, and the C-helix also provides a hydrophobic surface (on the underside of the hydrophobic groove) against which a variety of hydrophobic side chains can pack.

Recently, more sophisticated computational analyses of kinase conformational states and activation that use statistical analysis of kinase structures or simulation have been presented.^16,30^ It would be a significant step to combine these approaches with high-throughput crystallography to assess the conformational space accessible to kinase inactive states. Do some kinases have access to a wider range of conformations for a functional reason, and does this reflect the different regulatory mechanisms of these kinases? Furthermore, this analysis should be applied to a large number of kinases so that our knowledge is not biased toward a few models. This approach may be used to tackle the selectivity problem in kinase drug discovery by the prediction of specific inactive conformations of kinases that can be targeted by small-molecule inhibitors.

## Methods

### Protein expression and purification

The structure of the Nek2 kinase domain mutated at the activating threonine, T175A, has previously been determined.^12^ The mutant kinase domain was used because the wild-type kinase domain is insoluble and unstable. We reasoned that since the T175A mutant is stably expressed, the poor physicochemical properties of the wild-type protein are due to excessive kinase activity and resultant auto-phosphorylation. Co-expression of the wild-type kinase with lambda phosphatase improved the solubility properties, enabling the production of sufficient material for crystallization.

The cloning of Nek2-T175A in pET22b has been previously reported.^12^ Wild-type Nek2 was cloned into the NdeI/XhoI sites of pET30, giving rise to the same protein sequence as in pET22b, including an uncleavable C-terminal His_6_ tag. Lambda phosphatase was cloned into the NcoI/EcoRI sites of pCDF-Duet (Novagen). The coding sequence of the plasmids was confirmed by sequencing. Plasmids encoding either T175A-Nek2 or wild-type Nek2 and lambda phosphatase were transformed into CodonPlus RPIL *Escherichia coli* (Stratagene) and were cultured at 37 °C to an *A*_600_ of 0.5. The temperature was reduced to 18 °C for 30 min. Expression was induced using 1 mM isopropyl β-d-1-thiogalactopyranoside. Cells were harvested by centrifugation after 4 h and stored at − 80 °C. For purification, a 2-l cell pellet was resuspended in lysis buffer (50 mM Hepes, pH 7.5, 5 mM sodium phosphate, 300 mM sodium chloride, 5% glycerol and 20 mM imidazole) and was lysed by sonication. The lysate was loaded on a 1-ml HisTrap column (GE Healthcare), and Nek2 was eluted using a 20–250 mM imidazole gradient. Eluted Nek2 protein was purified to homogeneity using a 16/60 Superdex S200 HiPrep column (GE Healthcare) equilibrated in 50 mM Hepes, pH 7.5, 300 mM sodium chloride, 10 mM sodium phosphate, 5 mM dithiothreitol and 5% glycerol. T175A-Nek2 was treated with 40 μl of SAP (1 U/μl, USB) and 5 mM magnesium chloride over 2–3 days. Purified protein was concentrated to 5–8 mg/ml and was flash frozen in 10-μl aliquots. The protein was partially precipitated after 1 day at 4 °C. Protein that had been frozen crystallized readily, and the resultant crystals diffracted well.

### Crystallization of nucleotide-bound Nek2 wild type and T175A

Crystals of Nek2 were produced by vapor diffusion using the hanging-drop method. The well buffer used contained 2%–10% PEG (polyethylene glycol) 8000 and 50 mM Tris, pH 8.5, mixed 1:1 with the concentrated protein solution supplemented with 5 mM ADP and 5 mM magnesium sulfate for the ADP crystals, 5 mM ADP and 10% dimethylsulfoxide for the apo crystals and 5 mM ATPγS for the ATPγS crystals. Drops were immediately micro-seeded, and crystals grew to approximate dimensions of 50 μm × 20 μm × 5 μm in 2 days. Harvested crystals were briefly transferred to cryoprotectant (12% PEG 8000, 60 mM Tris, pH 8.5, and 28% ethylene glycol) and flash frozen in liquid nitrogen. We also produced apo-form crystals by soaking ADP crystals in well buffer solution over 24 h.

### Structure solution and refinement

X-ray diffraction data were integrated using Mosflm^31^ and scaled using SCALA.^31^ The structure of Nek2-T175A^ATPγS^ was solved by PHASER^31^ using the Nek2-T175A^SU^ model. Nek2^ADP^ and Nek2-T175A^Apo^ were solved using Nek2^ATPγS^ as the model. Structures were refined using Phenix^32^ and built using Coot.^31^ TLS refinement used a single group for Nek2-T175A^ATPγS^ and Nek2-T175A^Apo^, and eight groups were defined using TLSMD^33^ for Nek2^ADP^. The final models comprised residues 3–131, 138–166, 171–191 and 193–271 (+ 8-residue tag) for Nek2^ADP^, residues 3–131 and 138–271 (+ 8-residue tag) for Nek2-T175A^ATPγS^ and residues 3–131, 139–164, 168–189 and 192–271 (+ 8-residue tag) for Nek2-T175A^Apo^. Least-squares fitting used LSQKAB.^31^ Structure figures were produced using PyMOL (Warren L. DeLano, “The PyMOL Molecular Graphics System,” DeLano Scientific, San Carlos, CA, USA†). An active Cdk2 model shown is 1QMZ; inactive, 1B39; and Aurora-A/TPX2, 1OL5. Crystal structures of Nek2-T175A^ATPγS^, Nek2^ADP^ and Nek2-T175A^Apo^ were used.

### Mutagenesis and kinase assays

Mutations were introduced into the pRMCV-myc-Nek2A plasmid using the Gene Tailor™ Site-Directed Mutagenesis System (Invitrogen) and confirmed by DNA sequencing (Lark Technologies). For *in vitro* kinase assays, myc-Nek2A proteins were generated by coupled *in vitro* transcription–translation reactions using the TnT kit according to the manufacturer's instructions (Promega) and then immunoprecipitated with anti-myc antibodies and the immunoprecipitates used in kinase assays with β-casein as substrate, as previously described.^12^

### Accession codes

The coordinates of Nek2-T175A^ATPγS^, Nek2^ADP^ and Nek2-T175A^Apo^ and their associated structure factors have been deposited in the Protein Data Bank with accession codes 2W5B, 2W5A and 2W5H, respectively.

## Figures and Tables

**Table 1:** Summary of crystallographic analysis

	Nek2^ADP^	Nek2-T175A^ATPγS^	Nek2-T175A^Apo^
*Crystals*			
Space group	*C2*	*C2*	*C2*
Lattice constants			
*a*(Å)	99.47	99.60	99.74
*b* (Å)	57.10	57.06	56.96
*c* (Å)	80.21	80.80	80.61
β (°)	132.99	133.45	133.36

*Data collection*			
X-ray source	European Synchrotron Radiation Facility 14.2	European Synchrotron Radiation Facility 14.1	DIAMOND I03
Resolution range (Å) (highest-resolution shell values)	26.23–1.55 (1.63–1.55)	49–2.40 (2.53–2.40)	58.6–2.33 (2.46–2.33)
Unique reflections	45,663 (5161)	12,943 (1884)	12,969 (1453)
Completeness (%)	95.6 (74.5)	99.2 (99.2)	91.3 (72.3)
Multiplicity	3.6 (2.8)	2.5 (2.5)	3.7 (3.6)
*R*_merge_ (%)	4.8 (32.4)	9.7 (19.9)	10.8 (35.9)
*I*/σ(*I*)	15.8 (3.1)	3.9 (2.9)	6.1 (2.0)

*Refinement*			
Resolution range (Å)	24.74–1.55	49–2.40	58.6–2.33
No. of amino acids	266	271	263
No. of water molecules	355	166	120
No. of Mg ions	1	1	–
No. of Cl ions	–	1	–
No. of ethylene glycol molecules	4	–	–
Name of ligand bound to active site	ADP	ATPγS	–
*R*-factor (%)	17.21	18.3	19.5
*R*_free_^a^ (%)	19.01	24.5	25.4

*Ramachandran plot (%)*			
Most favored	91.5	90.9	91.4
Allowed	8.5	9.1	8.6
Generously allowed	0.0	0.0	0.0
Forbidden	0.0	0.0	0.0

a Free *R*-factor was computed using 5% of the data assigned randomly and is the same for all three structures.^27^

## Footnotes

† http://www.pymol.org
